# Band Gaps and Optical Properties of *RE*NiO_3_ upon Strain: Combining First-Principles Calculations and Machine Learning

**DOI:** 10.3390/ma16083070

**Published:** 2023-04-13

**Authors:** Xuchang Tang, Zhaokai Luo, Yuanyuan Cui

**Affiliations:** School of Materials Science and Engineering, Shanghai University, Shanghai 200444, China

**Keywords:** rare earth nickelates, epitaxial strain, machine learning, first-principles calculations

## Abstract

Rare earth nickel-based perovskite oxides (*RE*NiO_3_) have been widely studied over recent decades because of their unique properties. In the synthesis of *RE*NiO_3_ thin films, a lattice mismatch frequently exists between the substrates and the thin films, which may affect the optical properties of *RE*NiO_3_. In this paper, the first-principles calculations were employed to study the electronic and optical properties of *RE*NiO_3_ under strain. The results showed that with the increase in tensile strength, the band gap generally shows a widening trend. For optical properties, the absorption coefficients increase with the enhancement of photon energies in the far-infrared range. The compressive strain increases the light absorption, while the tensile strain suppresses it. For the reflectivity spectrum in the far-infrared range, a minimum reflectivity displays around the photon energy of 0.3 eV. The tensile strain enhances the reflectivity in the range of 0.05–0.3 eV, whereas it decreases it when the photon energies are larger than 0.3 eV. Furthermore, machine learning algorithms were applied and found that the planar epitaxial strain, electronegativity, volume of supercells, and rare earth element ion radius play key roles in the band gaps. Photon energy, electronegativity, band gap, the ionic radius of the rare earth element, and the tolerance factor are key parameters significantly influencing the optical properties.

## 1. Introduction

**Figure 1:**
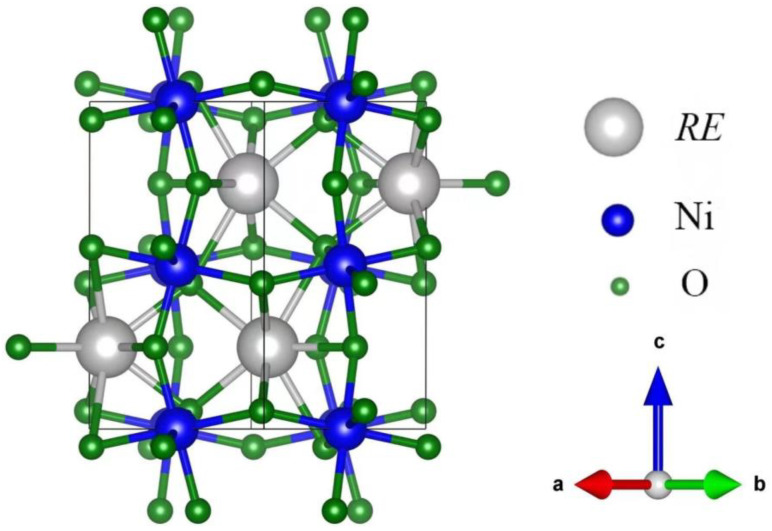
Geometric structure of *RE*NiO_3_.

Rare earth nickelates (*RE*NiO_3_) comprise a family of strongly correlated oxides with a distorted perovskite structure. In the atomic configurations of *RE*NiO_3_, the *RE* atoms are located in the center of the unit cell, and NiO_6_, with an octahedral structure, surrounds the *RE* atoms. The geometric structure is shown in Figure 1. Changing the *RE* types [1] or applying interface stress [2] could distort the atomic structure of *RE*NiO_3_, leading to the variations of its optical and electronic properties. This makes *RE*NiO_3_ applicable in tunable photonic devices [3], neuromorphic devices [4], and other applications [5,6,7].

The formation energy of *RE*NiO_3_ is positive, which means that bulk *RE*NiO_3_ is difficult to be synthesized through conventional strategies [8]. Currently, thin film deposition techniques, such as pulsed laser deposition (PLD) and molecular beam epitaxy (MBE), are the main methods used to obtain high-quality *RE*NiO_3_ films [9]. A mismatch frequently exists between *RE*NiO_3_ and substrates, which causes stress in the interface. For instance, Florine Conchon et al. [10] synthesized SmNiO_3_ layers onto SrTiO_3_ and LaAlO_3_ substrates through metal–organic chemical vapor deposition, proving that the planar tensile strain leads to the formation of oxygen vacancies. Ashvani Kumar et al. [11] observed the metal–semiconductor transition in PrNiO_3_ films deposited on SrTiO_3_, and attributed this transition to strain. Ai Ikeda et al. [12] synthesized *RE*NiO_3_ films by metal–organic decomposition. They found that epitaxial strain eliminated the occurrence of the metal–insulator transition in the PrNiO_3_ films on LaAlO_3_. In contrast, the NdNiO_3_ films on LaAlO_3_ exhibited a significant decrease in the temperature at which the metal–insulator transition occurred.

These reported papers indicate that the existence of strain causes oxygen vacancy [10] and the appearance of other phases [11], which influence the crystal structure and electronic structure of *RE*NiO_3_ thin films. As the band gap and optical properties are closely correlated with the electronic structures [13], the impact of strain on the band gaps and optical properties of *RE*NiO_3_ is still an open issue.

In addition, machine learning has emerged as a powerful tool for identifying patterns in high-dimensional data and can help to reduce the computational burden of materials discovery. By exploiting high-throughput databases and predicting material properties from existing data, machine learning can accelerate the exploration of novel materials.

In this paper, the band gaps and optical properties of *RE*NiO_3_ with different planar strains are studied using first-principles calculations and machine learning. We systematically investigate the effect of strain on the band gap and optical properties of *RE*NiO_3_. Our findings have significant implications for understanding the influence of strain on these materials and can guide future experimental work in this area. Moreover, the prediction model we develop can help costly first-principles calculations in predicting the properties of *RE*NiO_3_ under strain, thereby saving significant time and computing resources. Through this work, we aim to provide a deeper understanding of the underlying mechanisms governing the optical behavior of this material. This knowledge can serve as a foundation for further research on *RE*NiO_3_-based devices.

The layout of this paper is as follows: Section 2 provides details of the computational and modeling methods. Section 3.1 displays the band gaps calculated using the first-principles and the mathematical model built to describe the band gaps of *RE*NiO_3_. Section 3.2 presents the influence of epitaxial strain on the optical absorption coefficients and optical reflectivity. This section also introduces the models built to predict the optical properties of *RE*NiO_3_ with strains from −2.0% to 2.0%. Section 4 summarizes the results.

## 2. Methods

The calculations were conducted using the Vienna ab initio simulation package (VASP), which is a plane wave functional code [14,15,16,17]. The valence electron configurations are O-2s^2^2p^4^, Ni-3d^8^4s^2^, Pr, Nd, Sm, Gd, Pr, Lu, Dy, Ho, Y-6s^2^5p^6^5d^1^. The 4f electrons of the rare earth elements were regarded to be frozen in pseudopotentials [18]. The exchange correlation parts of the density functional were treated using the generalize gradient approximation (GGA) of Perdew–Burke–Ernzerhof (PBE) [19,20], and the potentials were the projector-augmented wave (PAW) type. The Hubbard parameter *U* was added to the PBE functional using the rotationally invariant approach of Dudarev et al. [21] in order to obtain strong on-site Coulomb repulsions among the electrons. In our work, the basic calculations were performed in the supercells with 4 × 4 × 1 primitive unit cells for *RE*NiO_3_. There were 80 atoms in each supercell.

The cut-off energy for the plane-wave basis was 520 eV. The parameters ensured that the convergence of the wave function was 1 × 10^−5^ eV and the geometry optimizations were fully finished until the Hellmann–Feynman force was less than 0.01 eV/Å. The geometry optimization employed a 3 × 6 × 2 Monkhorst–Pack *k*-point grid.

The planar strain of *RE*NiO_3_ was defined as:

(1) $$S_{x}=\frac{a-a_{0}}{a_{0}},S_{y}=\frac{b-b_{0}}{b_{0}}$$

The strains in the (100) and (010) directions were denoted as *S_x_* and *S_y_*, respectively, while *a_0_* and *b_0_* represented the initial lattice parameters, and *a* and *b* represented the lattice parameters under strain. According to experiments conducted by Torriss et al. [22], the strain range is from −0.26% to 2.7%. In the experiments of M. K. Stewart et al. [23], the range of strain is from −1.2% to 1.7%. The maximum strain reported in the work of Ai Ikeda et al. [8] is below 2.3%. Thus, the strains applied to each supercell in this work ranged from −2.0% to 2.0% based on these previous findings.

*RE*NiO_3_ has significant potential for applications in devices due to its unique phase-change properties, which can be facilitated by light. Deeper research into its optical properties, particularly its absorption coefficient and reflectivity, can provide valuable information for controlling phase transitions. For optical properties, dielectric functions (*ε*(*ω*)) were employed and the absorption coefficient (*α*(*ω*)) and reflectivity (*R*(*ω*)) were calculated by using following formulas:

(2) $$\alpha \left(\omega \right)=\frac{\omega }{c}{\left[2\sqrt{\varepsilon_{1}{\left(\omega \right)}^{2}+\varepsilon_{2}{\left(\omega \right)}^{2}}-2\varepsilon_{1}\left(\omega \right)\right]}^{\frac{1}{2}}$$

(3) $$R\left(\omega \right)={\left|\frac{\sqrt{\varepsilon_{1}\left(\omega \right)+i\varepsilon_{2}\left(\omega \right)}-1}{\sqrt{\varepsilon_{1}\left(\omega \right)+i\varepsilon_{2}\left(\omega \right)}+1}\right|}^{2}$$

Machine learning was adopted to build the models that predict the band gaps (*E_g_*), optical absorption coefficient (*α*(*ω*)), and reflectivity (*R*(*ω*)) [24,25,26,27]. For the modeling of the band gaps, many intrinsic parameters, including planar strain (*S*), relative atomic mass (*M*), electronegativity (*X*), volume of supercells (*V*), nickel ion radius (*R_N_*), oxygen ion radius (*R_O_*), rare earth element ion radius (*R_R_*), and tolerance factor (*t*) [28] were taken as initial feature descriptors. StandardScaler was used to pretreat the training data. By using the recursive feature elimination (RFE) feature selection strategy [29], the correlation between the target properties and feature descriptors were studied. We then calculated the importance score for each feature based on the coefficient in the function and its independence from other variables. Lasso regression (Lasso) was adopted to fit the selected descriptors. For the modeling of the absorption coefficient and reflectivity, a similar method was applied to build the models. This work preliminarily chose ten kinds of descriptors, i.e., photo energy (*E_p_*), planar strain (*S*), relative atomic mass (*M*), electronegativity (*X*), volume of supercells (*V*), band gaps (*E_g_*), nickel ion radius (*R_N_*), oxygen ion radius (*R_O_*), rare earth element ion radius (*R_R_*), and tolerance factor (*t*) [28]. RFE was used to pick up appropriate descriptors. K-Nearest Neighbors (KNN) regression, which uses the most similar K samples to predict the target value, was used to build the models. This model uses data on similar materials to predict the behavior of a new material under certain conditions because materials with similar compositions and conditions are located close to each other in the feature space. In other words, KNN leverages the similarity between materials in the feature space to make accurate predictions.

## 3. Results and Discussion

### 3.1. Band Gaps

**Figure 2:**
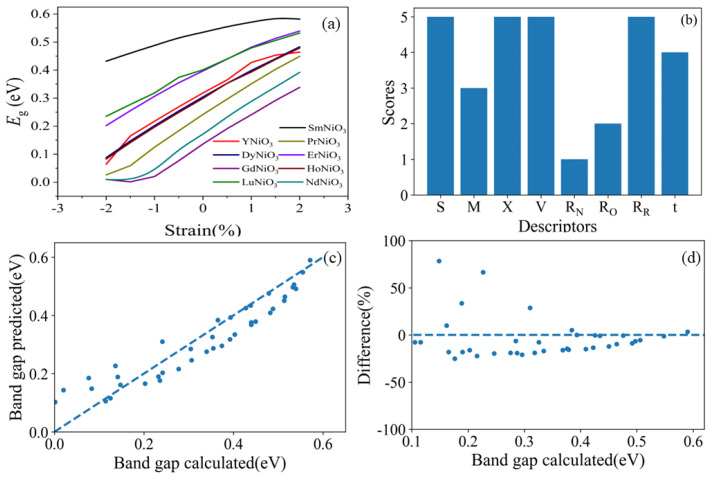
Band gaps of *RE*NiO_3_ (R = Sm, Y, Pr, Ho, Dy, Er, Lu, Nd, and Gd) upon strains ranging from −2.0% to 2.0% (**a**). Scores of the planar strain of *RE*NiO_3_ (*S*), relative atomic mass (*M*), electronegativity (*X*), volume of supercells (*V*), nickel ion radius (*R_N_*), oxygen ion radius (*R_O_*), rare earth element ion radius (*R_R_*), and tolerance factor (*t*) given by RFE (**b**). Comparison between the band gap calculated by VASP and the band gap predicted by this model (**c**). Difference between the band gap calculated by VASP and the band gap predicted by this model (**d**).

The band gaps are listed in Table 1 for each *RE*NiO_3_ (*RE* = Sm, Y, Pr, Ho, Dy, Er, Lu, Nd and Gd) upon strains ranging from −2.0% to 2.0%. Figure 2a shows that with the increase of tensile stress, the band gap generally shows a widening trend. It can be explained by the elongated Ni-O bonds upon tensile, which decreases the hybridization and overlap between O-2p orbital and Ni-3d orbital. As a result, the band gaps of *RE*NiO_3_ are widened under tensile [30]. When the thin films were subjected to compressive stress from the substrate, the Ni-O-Ni bond length was shortened and the bond angle was increased, resulting in an increase in the band width and a decrease in the band gap. Conversely, when the thin films were subjected to tensile stress from the substrate, the Ni-O-Ni bond length was elongated and the bond angle was reduced, leading to an increase in the band gap. Hu et al. [30] used pulsed laser deposition to synthesize SmNiO_3_ thin films and performed experimental characterization of the electrical transport properties. Their experimental results agree with our simulation results. Torriss et al. [22] also drew the following conclusion: “Actually, compressive strain broadens the bandwidth. In contrast, tensile strain causes the effective number of free carriers to reduce which is consistent with the d-band narrowing”. All of these factors suggest that the variation of the band gap with strain obtained from first principles calculation is consistent with the experimental results.

During the machine learning process, recursive feature elimination (RFE) was applied to score and rank the descriptors after preprocessing the data samples. Figure 2b shows that the planar strain of *RE*NiO_3_ (*S*), electronegativity (*X*), volume of supercells (*V*), and rare earth element ion radius (*R_R_*) are more suitable to be selected as the features in modeling. In order to verify the effect of RFE, we applied Lasso regression with all eight features, and the resulting coefficients are shown in Table 2.

It can be found that *M*, *V*, *R_N_*, *R_O_* all have small coefficients, which means that these four features have limited contributions to *E_g_*. This is why they were abandoned. At the same time, the tolerance factor has strong correlation to *R_N_*, *R_O_*, and *R_R_*, because the definition of the tolerance factor is $t=\frac{R_{R}+R_{O}}{\sqrt{2}\left(R_{N}+R_{O}\right)}$ [28]; this is why the score of the tolerance factor is a little smaller than *R_R_*. Overall, we thought the results of RFE were reasonable. Thus, we applied the features selected in the following work. Lasso was applied in building the model again. After fitting enough of a data sample, the band gap (*E_g_*) of *RE*NiO_3_ with different strains can be described as follows:

(4) $$E_{g}=3.17S-4.47X+0.003V-7.47R_{R}+10.9$$

Upon inspection of this model, the coefficient of determination (R^2^) is 0.88043. The mean squared error (MSE) and mean absolute error (MAE) are 0.00329 and 0.04852, respectively. Figure 2c presents a comparison of the band gaps calculated by VASP and predicted by the proposed model, indicating the proximity between the band gaps obtained from first-principles calculations and those predicted by the model. Figure 2d presents the errors of the predicted model, showing that the errors decrease with the increase in the large bands. In addition, the band gaps predicted by machine learning are slightly narrower than those calculated by first-principles calculations. It can be expected that the errors may be reduced with more training data input.

### 3.2. Optical Properties

**Figure 3:**
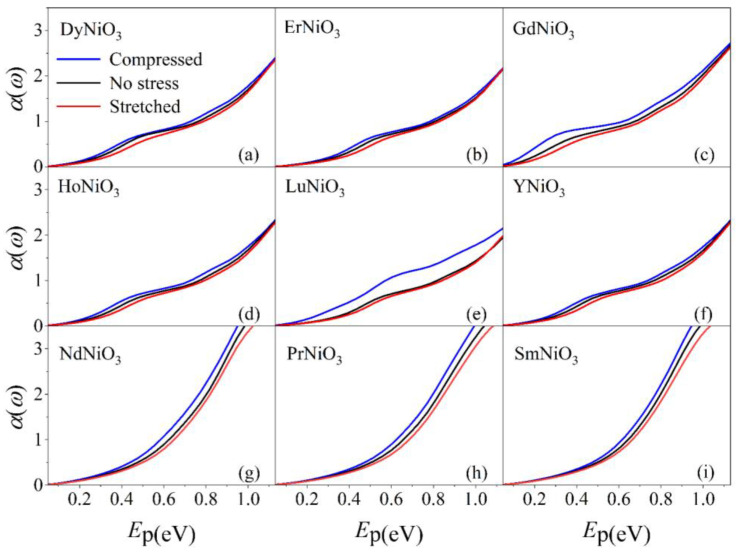
Absorption coefficients of DyNiO_3_ (**a**), ErNiO_3_ (**b**), GdNiO_3_ (**c**), HoNiO_3_ (**d**), LuNiO_3_ (**e**), YNiO_3_ (**f**), NdNiO_3_ (**g**), PrNiO_3_ (**h**), and SmNiO_3_ (**i**), where the absorption coefficient is given in 10^5^ cm^−1^.

**Figure 4:**
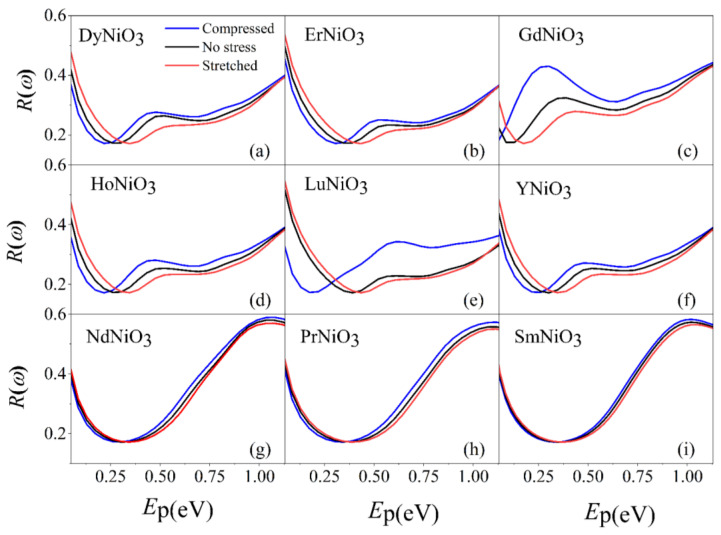
Reflectivity of DyNiO_3_ (**a**), ErNiO_3_ (**b**), GdNiO_3_ (**c**), HoNiO_3_ (**d**), LuNiO_3_ (**e**), YNiO_3_ (**f**), NdNiO_3_ (**g**), PrNiO_3_ (**h**), and SmNiO_3_ (**i**).

Figure 3 displays the absorption spectrum of *RE*NiO_3_ under different strains. In the far-infrared range (0.05 to 1.13 eV), there is an increase in absorption with the enhancement in photon energies. For *RE*NiO_3_ under different strains, Figure 3 indicates that the compressive strain increases the light absorption, while the tensile strain suppresses it. Figure 4 displays the reflectivity of *RE*NiO_3_ under different strains in the far-infrared range. There is a minimum reflectivity displayed around the photon energy of 0.3 eV. The tensile strain enhances the reflectivity in the range of 0.05–0.3 eV, whereas it decreases it when the photon energies are larger than 0.3 eV.

The optical properties of *RE*NiO_3_ under different strains can be explained as follows. The compression of *RE*NiO_3_ causes a decrease in the bond length of Ni-O-Ni and an increase in the bond angle. Conversely, the stretching of thin films leads to an increase in the bond length and a tightening of the bond angle. Changes in the lattice structure can significantly impact the band structure, as explained in Section 3.1. Specifically, modifications to the band structure can affect electronic transitions that result in the absorption or emission of light at varying wavelengths.

In the experimental side, Ardizzone et al. [31] observed that the in-plane low-frequency Drude weight doubled when the strain of LaNiO_3_ thin film transitioned from compression to stretch, which was caused by drastic changes in the electronic structure due to the alterations in the tilts and rotations of the NiO_6_ octahedra. Furthermore, they indicated that strain significantly impacts the interaction effects, resulting in an enhancement factor for optical effective mass ranging from approximately 1.5 to 3.0, demonstrating structural–electronic interdependence in transition–metal oxides. In research by Li et al. [3], SmNiO_3_ thin films were synthesized through physical vapor deposition and subsequent ultrahigh pressure annealing—a process that ensures the thermodynamically favored formation of the perovskite phase. The results have demonstrated that light can be tuned by using a hybrid structure consisting of plasmonic metasurfaces and thin-film nickelates, which means that nickelates have broad application prospects in the fields of optoelectronics, photonics, and information technology.

**Figure 5:**
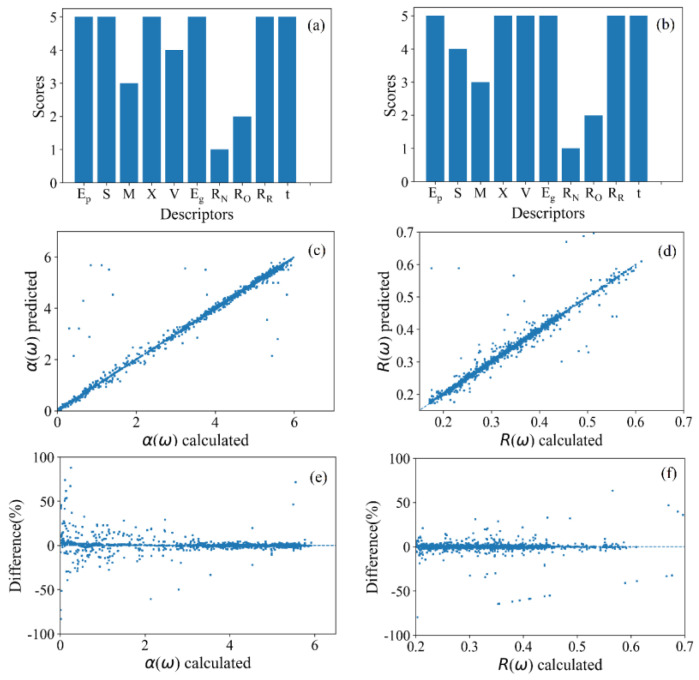
Scores of the absorption model descriptors evaluated using RFE methodology (**a**). Scores of the reflectivity model descriptors evaluated using RFE methodology (**b**). Comparison between absorption coefficient calculated by VASP and absorption coefficient predicted by this model (**c**). Comparison between reflectivity calculated by VASP and reflectivity predicted by this model (**d**). Difference between absorption coefficient calculated by VASP and absorption coefficient predicted by this model (**e**). Difference between reflectivity calculated by VASP and reflectivity predicted by this model (**f**).

To construct a prediction model for absorption coefficients and reflectivity, the energy of the photon (*E_p_*) and band gaps (*E_g_*) were screened as two extra descriptors in addition to the initial descriptors selected in Section 3.1. Then, RFE was applied to score and rank these descriptors. When modeling for the optical absorption coefficients, Figure 5a shows the scores of different descriptors, with the energy of each photon (*E_p_*), electronegativity (*X*), volume of supercells (*V*), band gap (*E_g_*), rare earth element ion radius (*R_R_*), and tolerance factor (*t*) scoring the highest. As a result, these six descriptors were used as the final descriptors for building the model. Figure 5c shows that nearly all of the predicted data are distributed around the diagonal, indicating the reliable prediction of our model. Figure 5e shows the errors that exist in the predictions made by the model. It indicates that the model has a better prediction effect for high absorption coefficient conditions. The MSE of this model is 0.06303, the MAE is 0.04826, and the R-squared value is 0.97787, suggesting the reliable prediction of *RE*NiO_3_ with strains from −2.0% to 2.0%.

In terms of describing the reflectivity of *RE*NiO_3_ thin films, the selected descriptors are listed in Figure 5b. The MSE and MAE of the reflectivity model were 0.00220 and 0.00833, respectively. Additionally, the R-squared value is 0.81284, which suggests that the model is effective for *RE*NiO_3_ thin films with strains ranging from −2.0% to 2.0%. Figure 5d displays a comparison between the reflectivity values calculated by VASP and those predicted by our model. The plot clearly illustrates that the calculated *R*(*ω*) values closely match the predicted *R*(*ω*) values. Based on the error plot shown in Figure 5f, it can be concluded that the predictive model is effective for most *RE*NiO_3_ thin film cases.

## 4. Conclusions

In this paper, the electronic and optical properties of strained *RE*NiO_3_ were studied using first-principles calculations and machine learning. The band gaps are narrowed under compression and the band gaps are widened under tensile. For the absorption coefficient of *RE*NiO_3_ with strain, the absorption coefficients increase with the enhancement of photon energies in the far-infrared range. The compressive strain increases the light absorption, while the tensile strain suppresses it. In the far-infrared range, the reflectivity spectrum shows a minimum around the photon energy of 0.3 eV. Tensile strain enhances reflectivity in the 0.05–0.3 eV range, but decreases it for photon energies greater than 0.3 eV. The expression of band gaps was constructed by machine learning was $E_{g}=3.17S-4.47X+0.003V-7.47R_{R}+10.9$, indicating that the epitaxial strain, electronegativity, volume of supercells, and rare earth element ion radius play key roles in affecting the band gaps. In addition, the models that can make precise predictions of the optical properties of strained *RE*NiO_3_ within a range of strains from −2.0% to 2.0% were developed. The models show that photon energy, electronegativity, band gaps, the ionic radius of the rare earth element, and tolerance factor are key factors that significantly influence the optical properties. The coefficient determination, mean squared error, and mean absolute error for the absorption coefficient model are 0.97787, 0.06303, and 0.04826, respectively. Similarly, the coefficient determination, mean squared error, and mean absolute error for the reflectivity model are 0.81284, 0.00220, and 0.00833, respectively.

## Figures and Tables

**Table 1:** Band gaps of *RE*NiO_3_ (*RE* = Sm, Y, Pr, Ho, Dy, Er, Lu, Nd, Gd) under different planar strains.

Strain	Sm	Y	Dy	Er	Lu	Ho	Pr	Nd	Gd
−2.0%	0.4318	0.0647	0.0879	0.2022	0.2354	0.0831	0.0263	0.0100	0.0101
−1.5%	0.4607	0.1652	0.1473	0.2553	0.2774	0.1414	0.0592	0.0122	0.0016
−1.0%	0.4882	0.2179	0.2018	0.3065	0.3186	0.1970	0.1253	0.0464	0.0204
−0.5%	0.5146	0.2692	0.2542	0.3548	0.3741	0.2491	0.1836	0.1145	0.0768
0.0%	0.5350	0.3187	0.3043	0.3971	0.4011	0.2991	0.2416	0.1721	0.1363
0.5%	0.5547	0.3652	0.3527	0.4395	0.4398	0.3530	0.2965	0.2324	0.1910
1.0%	0.5711	0.4275	0.3989	0.4819	0.4793	0.3934	0.3510	0.2882	0.2410
1.5%	0.5833	0.4534	0.4408	0.5131	0.5061	0.4381	0.4028	0.3400	0.2919
2.0%	0.5820	0.4642	0.4834	0.5394	0.5324	0.4788	0.4492	0.3922	0.3384

**Table 2:** Coefficients of each variable when using all eight features in Lasso regression.

S	M	X	V	*R_N_*	*R_O_*	*R_R_*	t
2.83	0.000447	−5.09	0.003	≈0	≈0	0.12	8

## Data Availability

The data presented in this study are available on request from the corresponding author.
